# Folic Acid and Leucovorin Have Potential to Prevent SARS-CoV-2-Virus Internalization by Interacting with S-Glycoprotein/Neuropilin-1 Receptor Complex

**DOI:** 10.3390/molecules28052294

**Published:** 2023-03-01

**Authors:** Ranko Škrbić, Maja Travar, Miloš P. Stojiljković, Dragan M. Djuric, Relja Suručić

**Affiliations:** 1 Department of Pharmacology, Toxicology and Clinical Pharmacology, Faculty of Medicine, University of Banja Luka, 78000 Banja Luka, The Republic of Srpska, Bosnia and Herzegovina; 2 Centre for Biomedical Research, Faculty of Medicine, University of Banja Luka, 78000 Banja Luka, The Republic of Srpska, Bosnia and Herzegovina; 3 Department of Microbiology, Faculty of Medicine, University of Banja Luka, 78000 Banja Luka, The Republic of Srpska, Bosnia and Herzegovina; 4 Institute of Medical Physiology “Richard Burian”, Faculty of Medicine, University of Belgrade, Višegradska 26/2, 11000 Belgrade, Serbia; 5 Department of Pharmacognosy, Faculty of Medicine, University of Banja Luka, 78000 Banja Luka, The Republic of Srpska, Bosnia and Herzegovina

**Keywords:** COVID-19, folic acid, in silico, in vitro, leucovorin, neuropilin-1, SARS-CoV-2

## Abstract

The interaction of the SARS-CoV-2 spike (S) glycoprotein receptor-binding domain with the host-cell ACE2 receptor is a well-known step in virus infection. Neuropilin-1 (NRP-1) is another host factor involved in virus internalization. The interaction between S-glycoprotein and NRP-1 has been identified as a potential COVID-19 treatment target. Herein, the effectiveness of folic acid and leucovorin in preventing contact between S-glycoprotein and NRP-1 receptors was investigated using in silico studies and then confirmed in vitro. The results of a molecular docking study showed that leucovorin and folic acid had lower binding energies than EG01377, a well-known NRP-1 inhibitor, and lopinavir. Two hydrogen bonds with Asp 320 and Asn 300 residues stabilized the leucovorin, while interactions with Gly 318, Thr 349, and Tyr 353 residues stabilized the folic acid. The molecular dynamic simulation revealed that the folic acid and leucovorin created very stable complexes with the NRP-1. The in vitro studies showed that the leucovorin was the most active inhibitor of the S1-glycoprotein/NRP-1 complex formation, with an IC_75_ value of 185.95 µg/mL. The results of this study suggest that folic acid and leucovorin could be considered as potential inhibitors of the S-glycoprotein/NRP-1 complex and, thus, could prevent the SARS-CoV-2 virus’ entry into host cells.

## 1. Introduction

Severe acute respiratory syndrome coronavirus 2 (SARS-CoV-2) is a virus with a crown-like envelope that is crucially involved in a process of viral infection. Its structure is very characteristic due to the presence of spike (S) glycoprotein, which is directly involved in the virus’ attachment to specific receptors on host cells [1]. The S-glycoprotein contains two fragments; the S1 subunit contains a receptor-binding domain (RBD), which binds to the host receptor angiotensin-converting enzyme 2 (ACE2) for initial docking, while the S2 subunit catalyzes the viral fusion with the host-cell membrane [2,3]. During the viral entry into the host cell, the S-glycoprotein is first pre-cleaved by furin, a membrane-bound member of the proprotein convertases family, at the S1 (amino acids 1–685) and the S2 (amino acids 686–1273) subunits. Subsequently, the S2 subunit is cleaved by the trans-membrane protease, serine 2 (TMPRSS2), at the S2′ site [4,5,6]. After the cleavage, the S1 subunit displays the C-end rule (CendR) polypeptide motif of the RRAR sequence (amino acids 682–685), which was recently found to be a docking area with neuropilin (NRP)-1 human cell surface receptor [7,8]. Therefore, the NRP-1 receptor serves as a co-receptor that promotes SARS-CoV-2 entry into the host cell. As a transmembrane glycoprotein receptor, NRP consists of two members, NRP-1 and neuropilin-2 (NRP-2), which regulate neurogenesis, angiogenesis, vascular permeability, and immune response, as well as tumor growth and vascularization [9,10]. Both receptor types have similar protein structures, with three extracellular domains, followed by a single transmembrane helix and a cytoplasmic tail [11]. The extracellular domain features three distinct domains. The a1a2 domain interacts with semaphorin 3 (SEMA3A), a secreted protein involved in neuronal development [9]. The b1b2 domain is homologous with the C-terminal domains of blood-coagulation factors V and VIII and vascular endothelial growth factor (VEGF) [9]. The c domain is homologous with meprin, A5, and µ-phosphatase (MAM), and it is responsible for complex assembly [12]. Several studies demonstrated that the SARS-CoV-2 S1-subunit directly binds to the b1b2 domain of NRP-1, also known as a coagulation-factor homology domain [7,8]. The binding of the S1 CendR motif, which is generated by the furin cleavage of S-glycoprotein, to NRP-1 mediates the internalization of CendR ligands through the process of endocytosis [8]. It was recently shown that NRP-1 stabilizes the S1 C-terminal region, stimulates the earlier separation of S2 from the S1 domain and, thus, increases viral infectivity [13]. Some proteins possess a CendR motif that allows them to bind the b1 domain of NRP-1, and VEGF-A is one of the most frequently studied ligands of NRP-1. Neuropilins and the VEGF receptors are involved in neural development, the function of axons and synapses, angiogenesis, and the permeability of blood vessels. Thus, the binding of the SARS-CoV-2 S-protein with the NRP-1 b1 domain may interfere with the receptor complex, which can deteriorate the signaling processes and host-cell functions [14]. It is well known that VEGF-A sensitizes nociceptor activity and initiates the sensation of pain [15]. The loss of olfactory function and reduced pain perception in SARS-CoV-2-infected patients could be explained by the effect of S-glycoprotein blocking the nociceptive VEGF-A/NRP-1 interaction. This connection between the SARS-CoV-2 S-protein and the NRP-1 receptor could additionally explain the longer-term effects of COVID-19 on the biological processes in the central nervous system and blood vessels. The inhibition of the VEGF-A/NRP-1 complex is considered as a potential target for inhibiting the SARS-CoV-2 virus’ entry [16]. In addition to interfering with the process of the virus’ internalization in host cells, several other targets during the process of viral replication, such as Mpro and RNA-dependent RNA polymerase, have been identified as promising therapeutic targets (RdRP). Several small ligands have also been identified as potential inhibitors of these enzymes. The Mpro inhibitors include camostat mesylate and galidesivir, while the RdRP inhibitors include remdesivir, arbidol, and favipiravir [17]. Researchers have become interested in purinergic receptors since it was recently found that they play a major role in making the central nervous system vulnerable to COVID-19 infection and in causing neurodegenerative diseases [18]. In their study, Pacheco and Faria focused on the significance of P2X7R receptors, whose activation during SARSCoV2 infection triggers the NLRP3 inflammasome and unfavorable inflammatory responses. An increased risk of comorbidities, such as diabetes and hypertension, has been associated with this pathway. Despite the fact that P2X7R and the NLRP3 inflammasome are emerging as therapeutic targets, more research on the role of the purinergic signaling pathway in COVID-19 is required, particularly for reducing the detrimental effects of the inflammatory responses associated with these diseases [19]. Computational studies have demonstrated that folic acid can prevent both the entry of SARS-CoV-2 into the host via ACE2 and subsequent replication, making it a good candidate for repurposing and further investigation [20]. According to the results of different studies, folic acid can inhibit both processes: SARS-CoV-2’s entry via ACE2 into the host and, subsequently, replication. This makes folic acid an attractive repurposing drug for further investigation. Several clinical studies showed that dietary intakes of vitamin C, folates, vitamin K, and fibers were associated with a lower susceptibility to SARS-CoV-2 infection [21,22,23]. It was also reported that pregnant women were 10 times less likely to be hospitalized for SARS-CoV-2 infection, which was ascribed to the folic acid supplementation during pregnancy and proposed to be a protective factor against SARS-CoV-2 infection [24]. Furthermore, it was shown that decreased serum folate levels were common among hospitalized patients with COVID-19 [25]. This was one of the reasons why we chose to test folic acid and leucovorin as potential inhibitors of the SARS-CoV-2 virus’ internalization. In this study, we hypothesized that folic acid and leucovorin interacted with the NRP-1 receptor-binding domain, thereby preventing the endocytosis of SARS-CoV-2. To test this hypothesis, computational methods such as molecular docking and molecular dynamics simulation were applied and then confirmed in vitro.

## 2. Results

### 2.1. In Silico Studies

**Figure 1:**
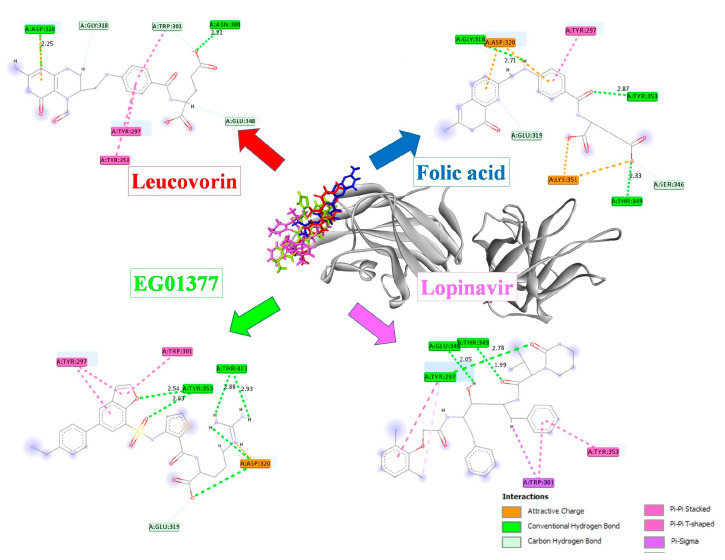
Tested compounds’ binding modes and two-dimensional illustration of interactions with NRP-1 residues.

The results of the molecular docking study showed that leucovorin and folic acid had the lowest binding energies, of −7.705 and −7.243 kcal/mol, respectively. The ascending binding-affinity order of these compounds and the amino acid residues for the pocket sites that were predicted to be involved in interactions with potential ligands, are presented in Table 1 and Figure 1.

**Figure 2:**
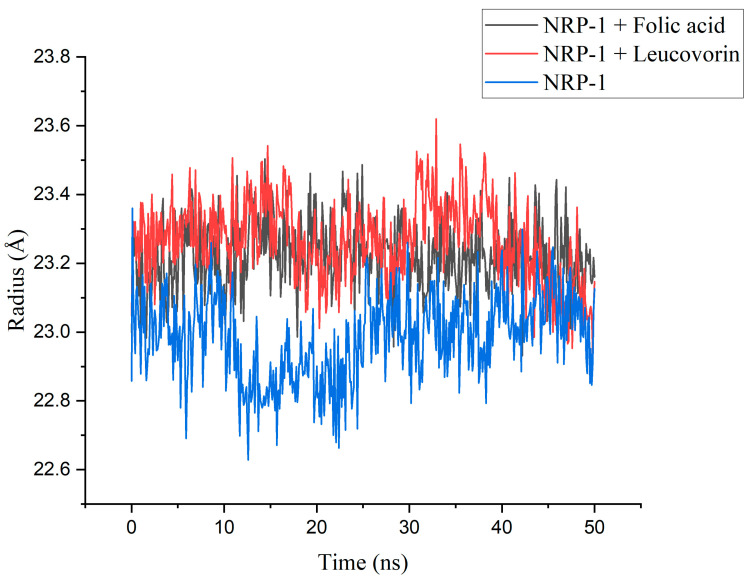
The radius of the gyration of NRP-1 without ligand and in complexes with folic acid and leucovorin.

The structural and dynamic behavior of the docked complexes was evaluated using MD simulation parameters, demonstrating their stability in a biologically relevant environment over time. We examined the stability of NRP-1 in association with the ligands by observing the changes in the radius of gyration (Rg) and root mean square deviation (RMSD) parameters. In addition, we examined and compared the flexibility of the NRP-1 in both bound and unbound states. The Rg trajectory pattern can reveal whether the protein’s secondary structure is stable under physiological conditions. Large trajectory deviations usually indicate an unstable structure, with low potential for complexing. The MD simulations revealed that the folic acid and leucovorin, when combined with the NRP-1, created very stable complexes with low trajectory oscillations in patterns that were quite similar (Figure 2). After 35 ns of simulation, a slightly higher oscillation was noticed for the leucovorin. The unbounded state of the NRP-1, on the other hand, showed more noticeable oscillation, especially during the simulation period between 10 and 25 ns, after which it remained steady until the completion of the simulation. However, in the unbounded state, the Rg parameter’s mean value for the NRP-1 was lower than in the complex with the folic acid and the leucovorin (Table 2).

**Figure 3:**
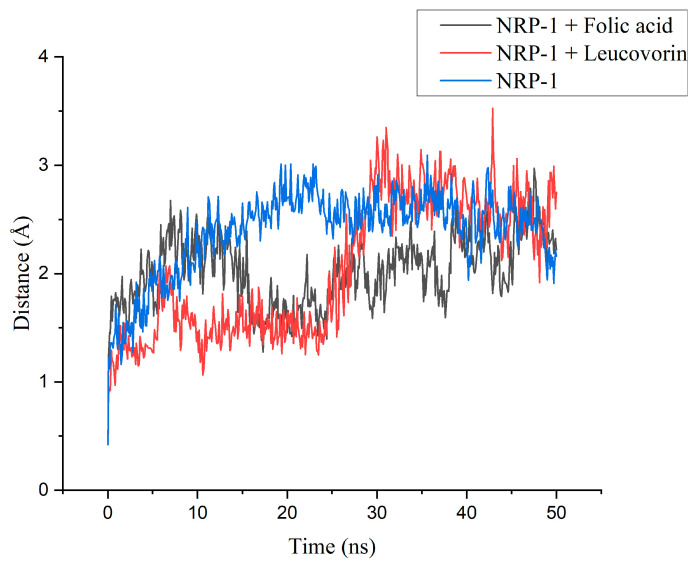
RMSD trajectories of NRP-1 without ligand and in complexes with folic acid and leucovorin.

By analyzing the RMSD trajectories (Figure 3), it was seen that the NRP-1 in an unbounded state achieved stability after 15 ns, whereas the trajectories of both complexes exhibited some deviations during the simulation. In the case of the folic acid, the variations were less pronounced. The complex structure was not jeopardized to a large extent, since the oscillations between the mean and maximum values did not exceed 1.5 Å for the complexes.

**Figure 4:**
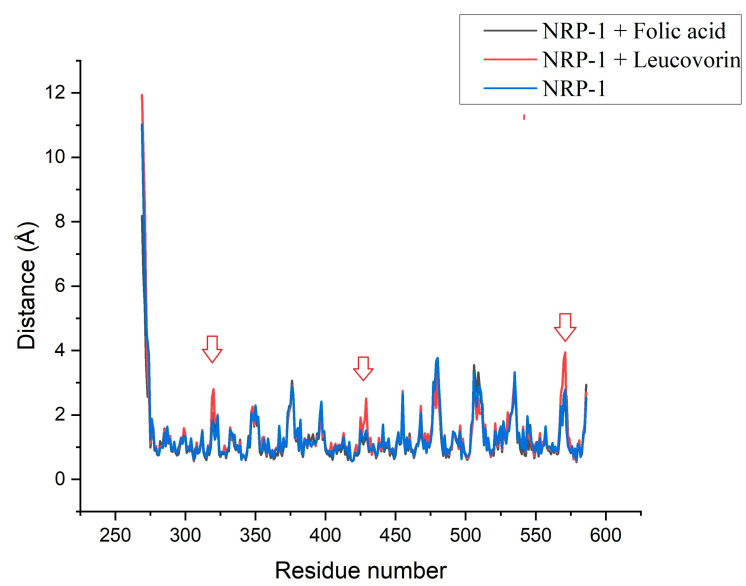
The RMSF graphs of NRP-1 without ligand and in complexes with folic acid and leucovorin. Red arrows indicate slight increase in flexibility.

Individual residue fluctuations were used to measure the flexibility of the protein–ligand complexes using RMSF trajectory analysis. The structural stability of a system is inversely proportional to its oscillations. The folic acid, leucovorin, and NRP-1 (in an unbounded state) all displayed similar patterns, showing that the ligand-protein interactions had no significant effect on the proteins’ structural flexibility. Only a slight increase in flexibility was detected in the NRP-leucovorin complex at the sites indicated by the red arrows in the Figure 4.

Finally, during a receptor-guided molecular docking simulation, significant hydrogen bonding and hydrophobic interactions were discovered. The structural stability of the leucovorin and folic acid at the active site was confirmed using MD simulation parameters.

### 2.2. In Vitro Study

**Figure 5:**
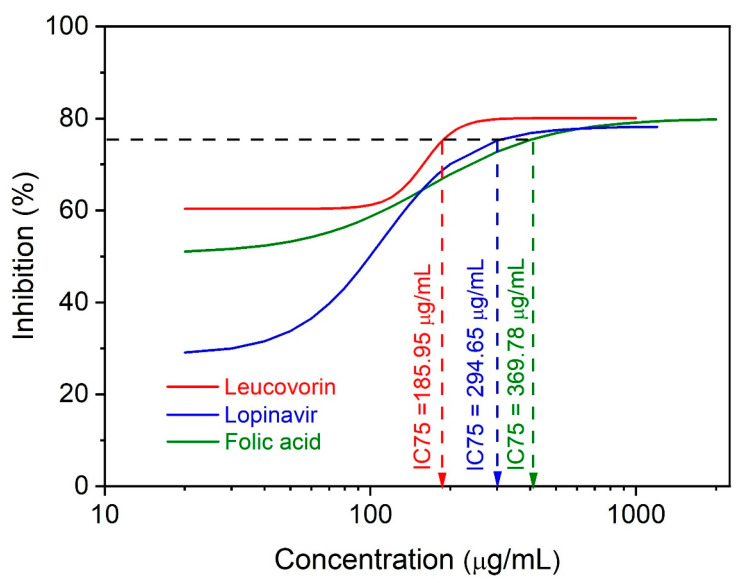
The concentration-inhibition response and IC_75_ values of in vitro-tested compounds against S-glycoprotein–NRP-1 interaction.

In the studied concentration range (50–1600 µg/mL), the folic acid, leucovorin, and lopinavir all showed considerable inhibitory efficacy (Figure 5). The 75% inhibitory concentration (IC_75_), indicating a larger amount of inhibition of the biological reaction, was less likely to arise by chance or through non-specific interactions. This is why the IC_75_ was used instead of the IC_50_ to minimize the likelihood of false-positive results. The leucovorin was the most active inhibitor, with an IC_75_ value of 185.95 µg/mL, as predicted by the in silico analysis. However, the in vitro study showed that the folic acid was a weaker inhibitor than the lopinavir.

## 3. Discussion

It has been recognized that the amino acid residues Tyr 297, Trp 301, Thr 316, Asp 320, Ser 346, Thr 349, and Tyr 353 are crucial for SARS-CoV-2 binding and nearly identical to the key interactions of VEGF-A with NRP1 [25]. A recent study showed that the binding of SARS-CoV-2 to NRP1 not only prevents the binding of VEGF-A, but also downregulates its signaling and function. As a result, the SARS-CoV-2 spike protein could significantly modify pain signaling by disrupting the VEGF-A/NRP1 complex [15]. This interaction has been considered as an attractive therapeutic antiviral target, since the specific molecules could successfully disrupt the SARS-CoV-2 NRP1 binding [26,27]. The urgent need for an effective treatment against the SARS-CoV-2 virus increased the number of in silico studies based on the repurposing technique to generate effective and safe drugs derived from drugs already approved for the treatment of other diseases. By controlling methylation in the promoter region of ACE2, folic acid influences the expression of ACE2. Consequently, this reduces the ability of spike protein, pseudovirus, and authentic SARS-CoV-2 that are inactivated to bind to host cells and further viral invasion. Additionally, a different molecular docking study suggests that folic acid has antiviral properties by inhibiting furin activity and acting on SARS-CoV-2 nucleocapsid phosphoprotein [28,29]. In the retrospective cohort study conducted between January 2020 and November 2022 on hospitalized adult patients with a polymerase chain reaction (PCR)-confirmed diagnosis of COVID-19, low folic acid levels were observed. However, there was no association between folate levels and disease severity or prognosis. This discovery is intriguing because it indicates that folate levels are associated with SARS-CoV-2 infection [25]. In the field of cancer biology, folic acid and neuropilin receptors have a connection that was previously described. Neuropilin receptors participate in cell signaling and axonal growth and migration guidance during development. They have been connected to tumor angiogenesis, which encourages tumor growth and metastasis. Additionally, it was shown that some cancer cells express more neuropilin receptors than others, which may lead to a more aggressive form of the disease. Although it has been reported that folic acid has an anti-angiogenic effect that is helpful for reducing tumor growth, folic acid supplementation is currently a controversial issue [30]. Since it is unclear whether folic acid prevents or promotes the development of cancer, it should be avoided in cancer patients, cancer survivors, and highly predisposed and susceptible people who are at risk of developing cancer [31]. Leucovorin, on the other hand, increased the effectiveness of other chemotherapy drugs in the treatment of cancer when used as an adjunct drug. The combination of bortezomib and leucovorin clearly demonstrated better therapeutic effects than the cytostatic alone [32]. Another serious condition associated with neuropilin and folic acid is thrombosis. Some studies have suggested that neuropilins may play a role in blood clot formation, while low levels of folic acid have been associated with an increased risk of thrombosis. This is thought to be due to the role of folic acid in maintaining proper homocysteine levels, as high homocysteine levels have been linked to an increased risk of blood clot formation [33,34]. The ideal folic acid dose should also be taken into account when examining these relationships because it has been demonstrated that high concentrations of folic acid have opposite effects on some markers in breast cancer patients [35]. In general, the recommended daily allowance (RDA) of folic acid for adults is 400 micrograms. However, some studies have used higher doses of folic acid, up to 5 milligrams per day, for the treatment of specific conditions, such as anemia or a history of neural tube defects in pregnancy [36]. In order to assess potential dose-dependent effects on this activity, additional in vivo studies of folic acid and leucovorin against SARS-CoV-2 must include both low and high concentrations of these substances. Based on positive clinical observations, these two well-known medicines were considered as potential inhibitors that could prevent CendR motif binding to the b1 domain of NRP1. The results of the molecular docking study showed that the leucovorin and folic acid both had very high binding affinity for NRP-1. The two medicines interacted with NRP-1 more effectively and with lower binding energies than the EG01377 and the lopinavir. Recently published computational screening data showed that the reference compounds, EG00229 and EG01377, have similar binding activities to NRP-1 [37]. However, the leucovorin and folic acid used in our study had a higher binding affinity to NRP-1 than these two reference compounds. Our data confirmed that conventional hydrogen bonds stabilized all the examined compounds in their energetically most favorable orientation. These interactions help to align the ligand in the proper orientation for binding and increase the stability of the complex by reducing the energy required to maintain the binding. Additionally, hydrogen bonds can also contribute to the specificity of the interaction by forming interactions with specific residues in the protein that are not present in other molecules. Two hydrogen bonds with Asp 320 and Asn 300 residues stabilized leucovorin, the molecule with the highest binding affinity for the target protein, while the folic acid complex was stabilized with the same type of interaction through Gly 318, Thr 349, and Tyr 353 residues. The residues Gly 318 and Glu 319 form the boundary of the binding pocket’s open region and were previously reported to interact with natural compounds [37]. In a recent study, the interactions of molecular-hydrogen-acceptor groups with the residues Thr 349 and Tyr 353 were also identified as critical for pharmacophore formation [16]. The residue Asp 320 is crucial for the coordination of the CendR motif’s terminal Arg, which is required for the interaction with S glycoprotein [38,39]. Except for lopinavir, all the compounds tested interacted with the Asp 320. Tetrahydropteridin and pteridin moiety, from the leucovorin and folic acid, respectively, were responsible for interactions with Asp 320. Hydrogen bonding was the primary difference between the leucovorin and the folic acid interactions; the folic acid’s interactions with the NRP-1 included weaker attractive charge interactions. However, the interactions with the Asn 300 residues were identified as significant NRP-1-contacting residues, making them possible NRP-1 inhibitors [40]. We can infer that both chemical complexes demonstrated good stability based on the variation between the minimal and maximal Rg values. Moreover, interactions with Asp 320 are crucial for interactions with VEGF C-terminal arginine [38]. Molecules that can interfere with VEGF/NRP-1 signaling could prevent the S-glycoprotein’s interaction with this complex, thereby not only reducing the viral entry, but also potentially preventing nociception, which is a very common symptom in SARS-CoV-2 infection. Although both compounds showed some potential as effective inhibitors, there were slight differences between the in silico and in vitro results. The in vitro studies showed that the leucovorin expressed the strongest inhibition of the S-glycoprotein–NRP-1 complex, with an IC_75_ value of 185.95 µg/mL, while the folic acid was shown to be a weaker inhibitor than the lopinavir. Other interactions beyond hydrogen bonding could possibly explain this finding. While the same number of hydrogen bonds was involved in the stabilization of both compounds, other, less attractive interactions, such as that of π-6 with Trp 301, may have prevailed for greater in vitro activity. During the stabilization of the EG01377-NRP-1 complex, interactions with the same residue were also detected. Kolarič et al. tested 20 compounds and two positive controls at a concentration of 100 µM in vitro using the same methodology as that employed in the present study [26]. The inhibition of the binding of the S-glycoprotein to the NRP-1 by the tested compounds ranged from 9.37% to 63.58%. All the compounds tested in this study were able to inhibit S-glycoprotein–NRP-1 contact at concentrations exceeding 70%, albeit at significantly higher concentrations. Folic acid and leucovorin have already been proposed as potential drugs against SARS-CoV-2 infection. In a recently published article, folic acid intake during pregnancy was suggested as a likely protective factor against SARS-CoV-2 infection [24]. Although the possible mechanism of this effect is still unclear, some in silico studies confirmed that folic acid can reduce viral replication. In one article, folic acid was shown as a potent inhibitor of furin endopeptidase [28], while in another report, folic acid was proposed as a potent inhibitor of SARS-CoV-2’s main protease [41]. The results of recently published in silico studies clearly showed that folic acid and its derivates, such as tetrahydrofolic acid and 5-methyl tetrahydrofolic acid, expressed a significant reduction in the major interacting residues between the spike protein and ACE2, indicating the potential of folic acid in the prevention of viral entry [42]. Similarly, leucovorin was also found to possess a favorable binding energy with SARS-CoV-2’s main protease suggesting a strong complex reaction with this enzyme [43]. In the relevant literature, no data related to the interaction of folic acid or leucovorin with the S-glycoprotein–NRP-1 complex have been reported so far. The results of this study confirm that folic acid and leucovorin could be considered as potential inhibitors of the S-glycoprotein–NRP-1 complex. This invites further in vivo assays focused on SARS-CoV-2 internalization.

## 4. Materials and Methods

### 4.1. In Silico Studies

#### 4.1.1. Molecular Docking Simulation

**Figure 6:**
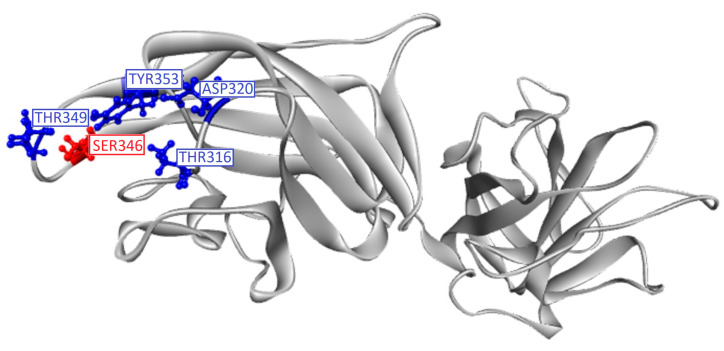
NRP-1 (PDB:2QQI) target with marked rigid (blue) and flexible (red) active-site residues.

The virtual screening receptor was chosen from the X-ray crystal structure of the b1b2 domains from human neuropilin-1 (PDBID:2QQI; 1.80 Å resolution). The clean module of the YASARA structure (version 20.12.24.W.64) was used to remove crystallographic waters, add polar hydrogens, and assign charges to titratable amino acids, followed by atom typing with the AMBER03 force field and geometry optimization using the steepest-gradient approach with 100 iterations. Docking studies were conducted with cubic grid box generated at a distance of 5 Å from selected amino acid residues. Vina algorithm was applied and Ser 346 amino acid residue was set to be flexible, while other residues were set to be rigid during docking calculations (Figure 6). Molecular docking studies were carried out to determine the binding affinity of folic acid and leucovorin, as well as their most stable spatial conformations during interactions with protein residues. The EG01377, a molecule with previously confirmed inhibitory effects against NRP-1 [44], and lopinavir, one of the drugs utilized in numerous studies for COVID-19 treatment [45], were also included in this investigation.

#### 4.1.2. Molecular Dynamics Simulation

Molecular dynamics (MD) simulations for the complex of the most stable folic acid and leucovorin complexes with b1b2 domain from human NRP-1 were also conducted using YASARA structure v. 20.12.24.W.64. Hydrogen-bond optimization and pKa prediction for the chosen pH (7.4) were part of the experimental setup [46]. The addition of NaCl ions (0.9 percent), cell neutralization, and energy minimization provided correct structure’s geometry. The MD simulation was run for 50 ns with AMBER14 force field. The setup used 298 K and one atmosphere for temperature and pressure values, respectively. Table S1 and Figure S1 depict the composition of the simulated systems, whereas Figures S2 and S3 in the Supplementary Materials depict the length of the simulation cells and the total potential energy of the systems.

### 4.2. In Vitro Study

Identification of molecules that interfere with the formation of the S-glycoprotein–NRP-1 complex was performed with the RayBio COVID-19 Spike-NRP-1 ELISA kit (RayBiotech Life, Inc. Peachtree Corners, GA, USA) according to manufacturer’s instructions. The assay methodology is depicted graphically in Figure S4 of the Supplementary Materials. This test is a rapid and sensitive method to characterize the binding affinity of the S-glycoprotein–NRP-1 complex in the presence of potential inhibitors.

#### 4.2.1. Serial Dilution Preparation

Serial dilution of the purified substances of folic acid, leucovorin, and lopinavir were prepared with 1x protein solution prepared according to manufacturer’s instructions. The volumes of 1.25 µL 100 × S1 protein concentrate and 125 µL 1 × assay diluents were prepared and quantities were multiplied by number of wells used for assay. All measurements of substances and controls were performed in duplicate to calculate average optical density (OD) absorbance across the replicate reagents.

#### 4.2.2. Assay Procedure

All reagents and substances were brought to room temperature (18–25 °C) before use. The RayBio COVID-19 Spike-NRP-1 Binding Assay Kit I contains a 96-well plate coated with recombinant NRP-1. Serial dilutions of folic acid, leucovorin, and lopinavir were added into the wells in the presence of recombinant spike S1 protein, according to the manufacturer’s instructions. Unbound S1 was removed with a wash step, and a mouse anti-S1 IgG detection antibody was added in order to bind to the S-NRP-1 complex. After washing, an HRP-conjugated anti-mouse secondary IgG was then applied to the wells in the presence of 3,3′,5,5′-tetramethylbenzidine (TMB) substrate. The HRP reacted with the TMB solution, producing a blue color that was proportional to the amount of bound S1. The HRPTMB reaction was stopped with the addition of the Stop Solution, resulting in a blue-to-yellow color change. The intensity of the color was then measured at 450 nm (BioTek 800 TS Absorbance Reader, Agilent, Santa Clara, CA, USA).

### 4.3. Data Analysis

The average OD absorbance across the replicate readings for each test reagent and controls was calculated. Data for the binding inhibition of each concentration of substances were compared with the OD data for the positive control (to which no test reagent was added). The percentage binding inhibition (BI%) was calculated according to manufacturer’s instructions.

## 5. Conclusions

Using molecular docking and molecular dynamics simulation studies, we confirmed that leucovorin and folic acid can be considered as good candidates for the inhibition of the interaction between the S-glycoprotein and the NRP-1 receptor. Both molecules showed a higher binding affinity to the NRP-1 than the reference compound, EG01377, and lopinavir. These in silico predictions were further confirmed by in vitro studies showing that leucovorin expressed the strongest inhibition of the S-glycoprotein–NRP-1 complex. The interactions of folic acid and leucovorin with NRP-1 described in this study demonstrated that, in addition to recognizing their ability to inhibit viral internalization, it is also useful to evaluate the therapeutic potential of these compounds during disease progression, particularly in the prevention of severe cardiovascular complications, bearing in mind the role of NRP-1 receptors as a target for the prevention and treatment of cardiovascular diseases. These interesting results need to be explored further and confirmed in cell-based biological evaluations. Bearing in mind that the COVID-19 pandemic is far from over, novel medicinal approaches are urgently needed.

## Figures and Tables

**Table 1:** Docking-simulation results.

Compound	Binding Energy (kcal/mol)	NRP-1-Contacting Residues
Leucovorin	−7.705	Asp 320*, Glu 348, Trp 301, Asn 300 *, Gly 318, Tyr 297, Tyr 353
Folic acid	−7.243	Glu 319, Gly 318 *, Ser 346, Asp 320, Lys 351, Thr 349 *, Tyr 297, Tyr 353 *
EG01377	−6.819	Glu 319, Thr 413 *, Tyr 297, Asp 320, Trp 301, Tyr 353 *
Lopinavir	−6.508	Glu 348 *, Thr 349 *, Trp 301, Tyr 297 *, Tyr 353

NRP-1: neuropilin-1; * hydrogen-bond interaction.

**Table 2:** Average, maximum, and minimal values for Rg and RMSD parameters during 50 ns of simulation.

Parameter		Folic Acid	Leucovorin	NRP-1
Rg	Mean	23.222	23.266	22.986
	Min	22.931	22.939	22.628
	Max	23.504	23.620	23.360
RMSD	Mean	2.025	2.044	2.385
	Min	0.425	0.440	0.419
	Max	2.973	3.525	3.092

## Data Availability

All data are available upon reasonable request.

## Supplementary Materials

The following supporting information can be downloaded at: https://www.mdpi.com/article/10.3390/molecules28052294/s1, Figure S1: A ray-traced picture of the simulated complexes of NRP-1 with (a) folic acid and (b) leucovorin. The simulation cell boundary is set to periodic; Figure S2: Simulation cell lengths [vertical axis] as a function of simulation time [horizontal axis] for (a) folic acid and (b) leucovorin; Figure S3: Total potential energy of the system [vertical axis] as a function of simulation time [horizontal axis] ] for (a) folic acid and (b) leucovorin; Figure S4: Graphical illustration of measuring the interaction between the Spike S1 domain and NRP1 in the presence of a potential inhibitor (RayBio® Spike-NRP1 Binding Assay Kit I); Table S1: Composition of the simulated systems.
