# Supplementary material for: Folic Acid and Leucovorin Have Potential to Prevent SARS-CoV-2-Virus Internalization by Interacting with S-Glycoprotein/Neuropilin-1 Receptor Complex
Source: Molecules. 2023 Mar 1;28(5):2294. doi: 10.3390/molecules28052294 (PMC10005443; doi:10.3390/molecules28052294)
Supplement: Supplementary file 1 [file molecules-28-02294-s001.zip › molecules-2188615-supplementary.pdf]

Supplementary materials

1. *In silico* studies

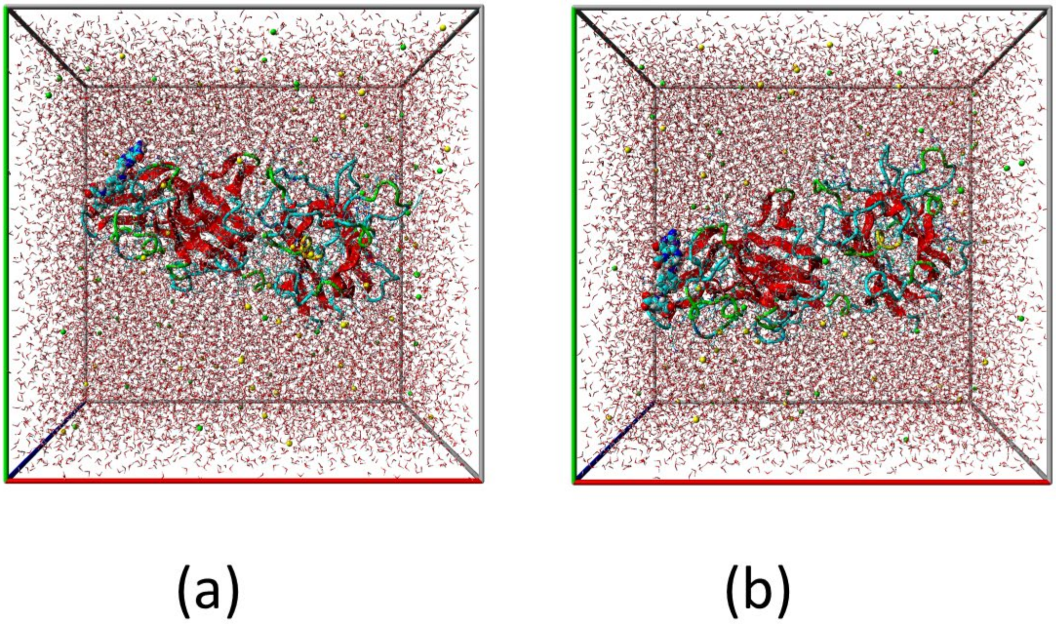

Figure S1. A ray-traced picture of the simulated complexes of NRP-1 with (a) folic acid and (b) leucovorin. The simulation cell boundary is set to periodic

Table S1. Composition of the simulated systems

	**Folic**	**Leucovorin**
**Type**	**Number**	
**Protein molecules**	1	1
**Protein residues**	318	318
**Protein atoms**	5024	5024
**Residue FNC with 55 atoms**	1	1
**Element Na**	58	55
**Element Cl**	59	56
**Water residues**	20799	19881
**Total number of atoms**	67587	64833

(a)

(b)

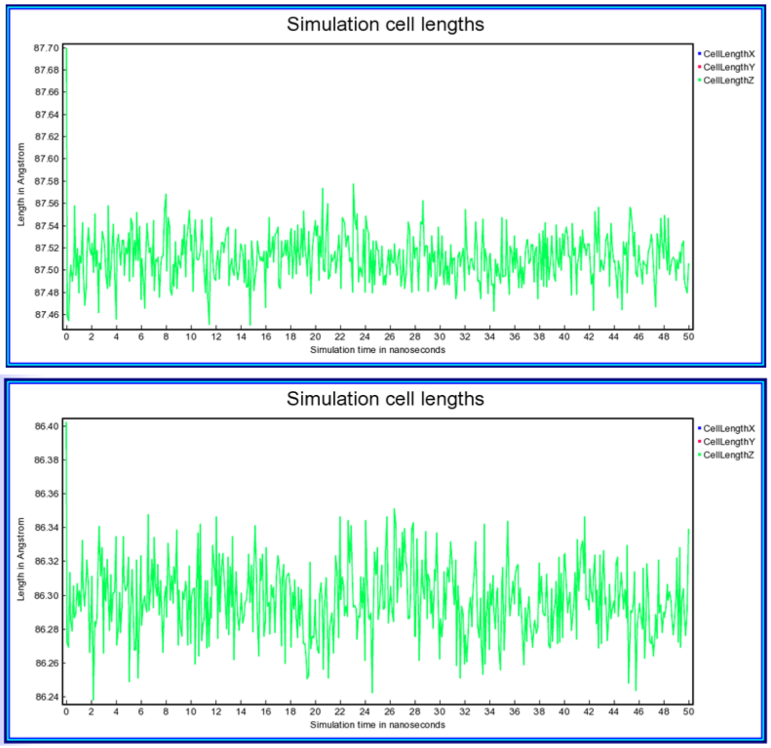

Figure S2. Simulation cell lengths [vertical axis] as a function of simulation time [horizontal axis] for (a) folic acid and (b) leucovorin

(a)

(b)

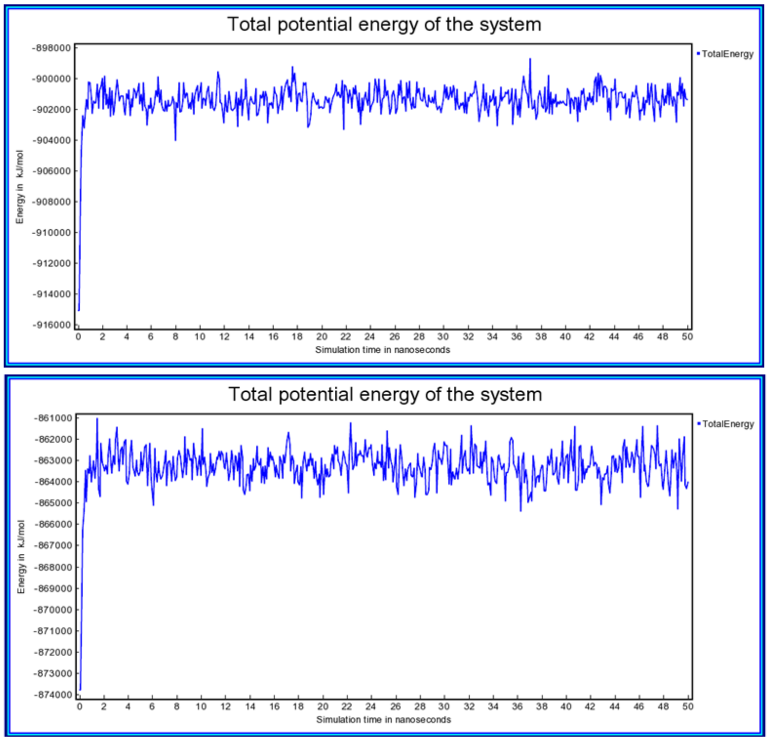

Figure S3. Total potential energy of the system [vertical axis] as a function of simulation time [horizontal axis]] for (a) folic acid and (b) leucovorin

2. *In vitro* study

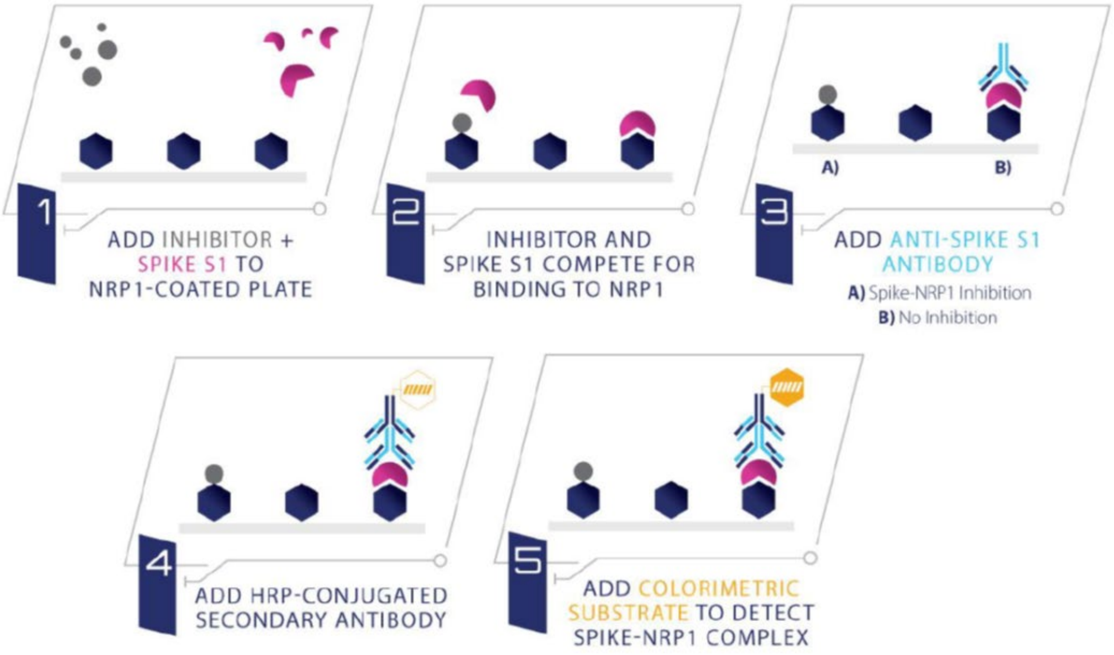

Figure S4. Graphical illustration of measuring the interaction between the Spike S1 domain and NRP1 in the presence of a potential inhibitor (RayBio® Spike-NRP1 Binding Assay Kit I).
